# Foliar Application of Boron Nanoencapsulated in Almond Trees Allows B Movement Within Tree and Implements Water Uptake and Transport Involving Aquaporins

**DOI:** 10.3389/fpls.2021.752648

**Published:** 2021-11-17

**Authors:** Juan J. Rios, Alvaro Lopez-Zaplana, Gloria Bárzana, Alberto Martinez-Alonso, Micaela Carvajal

**Affiliations:** Group of Aquaporins, Department of Plant Nutrition, Centro de Edafología y Biología Aplicada del Segura, CEBAS-CSIC, Campus Universitario de Espinardo, Murcia, Spain

**Keywords:** aquaporins (AQPs), boron, fertilization, nanoencapsulation, *Prunus dulcis*

## Abstract

Nanotechnology brings to agriculture new forms of fertilizer applications, which could be used to reduce environmental contamination and increase efficiency. In this study, foliar fertilization with nanoencapsulated boron (B) was studied in comparison to an ionic B (non-encapsulated) application in young B-deficient almond trees grown under a controlled environment. B movement within the plant in relation to the leaf gas exchange, water relations parameters, and root hydraulic conductance was measured. Also, the expression of aquaporins (AQPs) [plasma membrane intrinsic protein (PIP) and tonoplast intrinsic protein (TIP)] was studied in relation to water uptake and transport parameters to establish the effectiveness of the different B treatments. The obtained results were associated with a high concentration of observed B with nanoencapsulated B, provided by the higher permeability of carrier nanovesicles, which allowed B to reach the cell wall more efficiently. The increases in water uptake and transport obtained in these plants could be related to the role that this element played in the cell wall and the relationship that it could have in the regulation of the expression of AQPs and their involvement in water relations. Also, an increase in the expression of PIPs (mainly PIP2.2) to the applied nanoencapsulated B could be related to the need for B and water transport, and fine regulation of TIP1.1 in relation to B concentration in tissues provides an important feature in the remobilization of B within the cell.

## Introduction

At present, the use of nanotechnology-based methods has increased the scientific development of multidisciplinary strategies and their usefulness (Moradi et al., 2020). They could be used for agricultural applications such as fertilization. Because of the limited resources and an increase in human population, agriculture has to become more efficient for maintaining and even increasing the levels of production that will be needed in the future (Zhang et al., 2015). In this sense, soil chemical fertilizers have provided a good solution for maintaining production, but the pollution problems derived from the massive application of this kind of fertilization have provoked to result in the shifting of fertilization methods, with new methods such as a foliar application being currently investigated (Zulfiqar et al., 2019). The foliar application of nutrients has been defined as an eco-friendly method (Rios et al., 2020), but its efficiency has not reached the desired levels desired. Currently, the power of nanoparticles as nutrient carriers is being studied. Thus, according to the mineral they could transport, these novel fertilizers are classified as micronutrient or macronutrient nanofertilizers (Zulfiqar et al., 2019). Although the interest in this research field is very high, studies on this matter have been very limited in the past few years. Some of them have been conducted at our laboratory, where it was demonstrated that proteoliposomes from natural membranes could be a perfect carrier (Yepes-Molina et al., 2020), and they could be more effective fertilizers if microelements were nanoencapsulated than when compared to the free application of different salts (Rios et al., 2019). In this regard, a recent work carried out by Rios et al. (2020) showed that nanoencapsulation increased the absorption of certain elements through leaves.

Almond trees (*Prunus dulcis* L.) are the most important fruit trees in agriculture. Also, almond is an important agricultural product worldwide because it is highly consumed by humans (Barreca et al., 2020). Almond trees have been cultivated in poor soils and low rain-fed areas, which have consequently resulted in poor harvests in these areas. Currently, the commercialization of almonds has increased profits in the market, and the farmers’ interest in this crop has grown (Martinez-Gomez and Sánchez-Pérez, 2008). However, the low level of mineral nutrient in soils where it is usually cultivated, make necessary the use of intensive fertilization. Therefore, fertilization has to be efficient, economically viable, and environmentally sustainable. In this way, soil fertilization has been demonstrated to be poorly efficient in almond trees due to stressful environmental conditions where they are grown (Arrobas et al., 2019).

Boron (B) is a micronutrient that is involved in numerous cell and structural functions, such as cell structure, biosynthesis and lignification, cell elongation, and membrane permeability (Lewis, 2019). B deficiency usually occurs during the vegetative stage of plants in the soils that are poor in organic content and regions with low precipitation (Kitir et al., 2019). Therefore, it has to be externally supplied. However, as B is a micronutrient with limited mobility throughout the plant (Brown et al., 2002; Miwa and Fujiwara, 2010), the foliar application of B should have a local effect on the plant, but its main use on fruit trees, such as almond, has failed to increase the nutritional status of the crop (Arrobas et al., 2019).

In this regard, B is transported through plants and plant cells through the flow of water, with the transpiration and the physiological state of the plant being very important for this (Miwa and Fujiwara, 2010). The amount of water used by almond trees is determined by weather and can thus be influenced by humidity, temperature, and the time of day. Therefore, its use can be very restricted due to the stressful environmental conditions that are normally found in an orchard almond. In this way, almond trees grown under water stress in the leaf expansion stage have been shown to have a reduced vegetative growth as this is related to canopy growth and size (Lampinen et al., 2010). In addition, if the water loss due to transpiration exceeds the amount of water taken up by the roots, the tree can exhibit stress responses, starting with stomatal closure. This will produce a reduction in gas exchange, with subsequent decreases in the rates of photosynthesis and the production of carbohydrates (Morais et al., 2020), limiting the amount of energy available for vegetative growth, but also the transport of mineral nutrients, such as B, that move through the flow of water.

The processes of water uptake and transport in plants are associated with the presence and opening state of aquaporins (AQPs) as they transport water freely across the main cell membranes in response to changes in osmotic or hydrostatic potentials (Wang et al., 2019). AQPs can be classified into five families depending on their cellular membrane localization and amino acid sequence. Thus, they are classified as plasma membrane intrinsic proteins (PIPs), located in the plasma membrane; tonoplast intrinsic proteins (TIPs), in the tonoplast; nodulin26-like intrinsic proteins (NIPs), mainly located in the plasma membrane; small basic intrinsic proteins (SIPs), only identified in the endoplasmic reticulum to date; and the uncharacterized X-intrinsic proteins (XIPs) (Martinez-Ballesta and Carvajal, 2014). Therefore, the apparent simplicity of AQPs is, in fact, very complex due to a large number of families, isoforms within each family, and the specificity of some of them. Indeed, it has been reported that some AQPs from PIPs, TIPs, and NIPs are able to transport B (Dordas et al., 2000; Kato et al., 2009; Bárzana et al., 2014) and some of the NIPs have been characterized specifically as B transporters mainly under B deficiency (Takano et al., 2006; Tanaka et al., 2008). In addition, the expression of some isoforms was shown to increase under B-deficient conditions, indicating their important roles in B translocation (Barzana et al., 2020). However, very few studies have been performed in relation to B nutrition and the expression of AQPs that transport water (PIPs and TIPs) although some studies have revealed the connection between both processes (Bastías et al., 2004) and the movement of B through the flow of water. In this regard, some PIP isoforms have been shown to be regulated by B availability (Kumar et al., 2014; Mosa et al., 2016), and TIPs have been associated with B compartmentalization and posterior remobilization from vacuoles (Pang et al., 2010; Bárzana et al., 2014). To date, no studies have been performed on *P. dulcis* AQPs although their influence on improving the yield production of this crop may be crucial.

Foliar fertilization and the development of new nanofertilizers are currently being studied for enhancing the concentration of elements in plant tissues. However, the physiological responses of plants to the use of nanofertilizers have been analyzed in very few studies. Therefore, the aim of this study was to investigate the effect of B nanofertilizers in a foliar application on almond trees. For this, the effectiveness of application, the movement within the plant, and the response to leaf gas exchange, water relations parameters, and root hydraulic conductance, which were associated with AQP (PIP and TIP) expression, were studied.

## Materials and Methods

### Experimental Design and Culture Conditions

Clones of the *P. dulcis* L. variety Avijor 3 weeks old were acquired from Agromillora Iberia S.A. (Barcelona, Spain). The plants were grown for 15 days on a substrate. Then, they were transferred to hydroponic conditions in 16-L containers (4 trees in each of the 16 containers, with a total of 64 trees) filled with Hoagland’s solution, pH 5.5. The solution was continuously aerated and was changed every week. The composition of the solution was: 6 KNO_3_, 4 Ca (NO_3_)_2_, 1 KH_2_PO_4_, and 1 MgSO_4_ (mM), and 25 H_3_BO_3_, 2 MnSO_4_, 2 ZnSO_4_, 0.5 CuSO_4_, 0.5 (NH_4_)_6_Mo_7_O_24_, and 20 Fe-EDDHA (μM). The plants were grown under controlled conditions in a growth chamber, with a cycle of 16 h of light and 8 h of darkness, with temperatures of 25 and 20°C, and relative humidity of 70 and 80%, respectively. The photosynthetically active radiation (PAR) was 400 μmol m^–2^ s^–1^, provided by fluorescent tubes (Philips TLD 36 W/83, Jena, Germany and Sylvania F36 W/GRO, Manchester, NH, United States) and metal halide lamps (Osram HQI, T 400W, Berlin, Germany).

After 10 days, deficiency treatments were applied: 4 containers with full nutrient solution continued, and 12 containers with 0 μM of B and not applying H_3_BO_3_ in the Hoagland’s solution. The trees were grown under these conditions for 15 days. After these 15 days, the experiment was divided into two different sets with eight containers each (two for controls and six for treated trees).

In one set of experiments (S0), the foliar treatments were applied to only three fully expanded leaves from trees (avoiding those proximal to the meristematic apex) that were grown in B deficiency of four of the containers (four trees per container). The applied treatments were (i) B nanoencapsulated at 0.04% (from H_3_BO_3_) obtained according to Rios et al. (2020) applied to two containers and (ii) free B at 0.04% (from H_3_BO_3_) applied to the other two containers. In control and deficient plants (two containers each), water was applied. The treatments were applied to the abaxial side of the leaves of each plant using a spray with a surfactant (polyether-modified-polysiloxane) at 0.1%. Also, and according to the results shown by Rios et al. (2019), the nanovesicles themselves did not produce any effect on the plant. Two hours after the foliar treatment application, gas exchange parameters were measured. In addition, the microscopy analysis of penetrability was determined. Leaves (treated and non-treated) were collected for measuring osmotic potential, mineral analysis, and AQP expression. In total, S0 had: 6 containers × 4 trees × 3 treated leaves × 3 non-treated leaves.

In another set of experiments (S1), the treatments (i and ii) were applied to two containers to all fully expanded leaves that appeared and grew with B deficiency. In this experiment, also the treatments were applied to avoid the leaves proximal to the meristematic apex. In total, S1 had: 6 containers × 4 trees × 3 treated leaves, or 3 non-treated leaves.

For this experiment set (S1), the measurements and sample collection were done after 7 days from a foliar treatment. Gas exchange parameters, leaf area, root hydraulic conductance, leaf water relations, B concentration in tissues and cell wall extractions, and AQP expression were determined in the treated leaves and meristematic leaves. All measurements and sample collection were carried out for 4 h at the middle of the photoperiod. The foliar treatments were applied on the same day at the same time in S0 and S1.

An earlier test was performed for checking the effect of an empty nanoencapsulation system. The fact that there was no effect on the gas exchange parameters of leaves (Supplementary Table 1). Therefore, in the rest of the experiments, the treatment using a nanoencapsulation system was not used.

### Relative Growth of Meristematic Leaves

The relative growth of meristematic leaves was measured as an increased leaf area in the set (S1). Meristematic leaves, four per plant, were selected previously for treatment application, their outline was drawn on a sheet of paper, and the sheet was scanned later. After 7 days of growth, the selected leaves were collected and photographed. The images and scans were treated using the ImageJ program to measure the area of the leaves.

### B Concentrations

For an ionic analysis, the leaves were washed well with distilled water and lyophilized. Finely ground samples of lyophilized leaves were digested in a microwave oven (CEM Mars Xpress, Matthews, NC, United States) by HNO3:HClO4 (2:1) digestion. The elements were detected by an inductively coupled plasma (ICP) analysis (Optima 3000, PerkinElmer, Norwalk, CT, United States).

### Study of Fluorescein Diacetate Delivery From Vesicles and *in vitro* Leaf Penetration by Vesicles

Fluorescein disodium salt (FNA) (Sigma Aldrich, Madrid, Spain) at 50 μg ml^–1^ was encapsulated in vesicles (0.1% protein, w/v). For the encapsulation, FNA was added to vesicles, shaken vigorously, and washed in phosphate buffer [0.33 M sucrose, 5 mM potassium phosphate (pH 7.8)] by centrifugation at 100,000 × g for 35 min. The pellet was resuspended to obtain the nanoencapsuled solution according to Rios et al. (2020). The solution was sprayed on the almond leaves as previously mentioned. After 2 h, the leaves were thoroughly washed and dried to clean their surfaces. Finally, the leaf sections were observed using an inverted confocal laser scanning microscope (CLSM) (LEICA TCS-SP2, Leica Microsystems, Wetzlar, Germany) equipped with a UV/visible light laser. FNA was excited at 460 nm and detected at 515 nm. Whole-leaf layer images were taken to create a three-dimensional (3D) reconstruction from the leaf and vesicle penetration.

### Cell Wall Isolation

The cell wall was isolated according to Cassab et al. (1985). Approximately 10 g of fresh leaves from S1 were finely chopped and vacuum-infiltrated with 40 ml of solution that contained 5 mM Tris–HCl and 0.25 M sucrose. The infiltration solution was adjusted to pH 7.2, and 1 mM Mg(AcO)_2_ was added. Later, the material was homogenized using a pestle, mortar, and sea sand, and filtered through a nylon cloth with a pore diameter of 240 mm. Afterward, the filtrate was centrifuged for 15 min at 1,000 × g, at 4°C (J2-21 centrifuge, Beckman, Palo Alto, CA, United States). The supernatant was discarded, and the obtained pellet was resuspended in 2 ml of 5 mM Tris–HCl pH 7 buffer containing Triton X-100 (1% v/v) and m-octanol (0.2% v/v). This mix was centrifuged for 15 min at 1,000 × g, at 4°C. This step was repeated two times, and the resulting pellet was washed with 2 ml of 50 mM Tris–HCl, pH 7.2 buffer, and centrifuged again. The resulting pellet was an isolated cell wall, and ionome analysis using ICP was performed.

### Root Hydraulic Conductance

Plants from the S1 experiment were used to measure the root hydraulic conductance (*L*_*h*_) by pressurizing the roots using a Schölander chamber (Jackson et al., 1996) as described by Fernández-García et al. (2002). The sap flow (*J*_*v*_) was expressed in mg g^–1^ root fresh weight (RFW) h^–1^ and plotted against pressure (MPa), with the slope being the *L*_*h*_ value in mg g^–1^ (RFW) h^–1^ MPa^–1^. The measurements were made in the middle of the photoperiod.

### Leaf Water Relations

Leaf water relations, such as water potential (Ψ_*w*_) and osmotic potential (Ψ_π_), were measured according to Fernández-García et al. (2002). However, a difference between Ψ_*w*_ and Ψ_π_ was calculated to obtain the turgor potential (Ψ_*t*_).

### Gas Exchange Parameters

Gas exchange parameters—transpiration, stomatal conductance, assimilation rate, and internal carbon dioxide (CO_2_) (CI)—were measured in a fully expanded leaf grown under B deficiency and in the same growth area for the control treatment of each plant with an LI-6400 portable photosynthesis system (LI-COR, Inc., Lincoln, NE, United States), in a total of three leaves per plant and per treatment. These measurements were made in S0 plant leaves, where B was applied and not applied, and in apex leaves 2 h after B foliar application. For S1, this was performed 7 days after B application at the same time as S0. All the measures and sample collection were carried out always at the same time for 2 h in the middle of the photoperiod. In addition, water use efficiency (WUE) was calculated using the assimilation/transpiration ratio.

### RNA Extraction and Complementary DNA Synthesis

Total RNA was extracted using the NucleoSpin^®^ RNA Plant and Fungi kit (Macherey-Nagel, Düren, Germany) according to the protocol of the manufacturer. The RNA integrity was measured *via* RNA electrophoresis, and the RNA concentration was quantified using a NanoDrop^TM^ One (Thermo Fisher Scientific, Waltham, MA, United States). The remaining DNA was removed by using the DNAase I RNase-free kit (Ambion, Applied Biosystems, Austin, TX, United States). Contamination with DNA was discarded in the sample sets by running a control PCR with aliquots of the same RNA that had been subjected to the DNase treatment but not to the reverse-transcription step, using an Eppendorf Mastercycler^®^ Personal (Eppendorf AG, Hamburg, Germany). The High-Capacity cDNA Reverse Transcription kit MultiScribe^TM^ (Applied Biosystems, Austin, TX, United States) was used to synthesize complementary DNA (cDNA) from 2 μg of total RNA. The synthesis was made with heat denaturation of the RNA according to the instructions of the manufacturer.

### Phylogenetic Tree

As no previous studies were conducted on almond AQPs, we first did a general search in the National Center for Biotechnology Information (NCBI) database for all the sequences of PIPs and TIPs (AQPs) available for *Prunus* sp., the sequences were aligned by using the Clustal Omega online program^1^ and shown as a phylogenetic tree. For this, MUSCLE was used to align the sequences, and the NJ method (with 1,000 bootstrap replications) was used to build a tree, all with MEGA X. The phylogenetic tree design was performed using the online tool ‘‘Interactive Tree Of Life’’ (iTOL).^2^

The general AQP groups were represented, and within each group of PIPs and TIPs, different branches with the 98–100% homology between the AQPs of the different *Prunus* sp. were identified. AQPs from *P. dulcis* were also detected in the NCBI database as an unconfirmed bioinformatic prediction, which was correlated with the different branches of *Prunus* sp., one in each subgroup. Based on this, the AQPs were renamed by homology with all the other *Prunus sp*. AQP sequences in the NCBI database (Supplementary Figure 1 and Supplementary Tables 2, 3).

### Primer **Design** and **Real**-Time Q*uantitative PCR* **Analysis**

Specific primers were designed for the complete PIP and TIP genes of *P. dulcis*. The primer sequences of the 15 *P. dulcis* AQP genes are shown in Supplementary Table 2. The primer design was carried out manually, meeting the specific requirements imposed by a high homology of the sequences used and the technique used for the analysis. Virtual analysis of melting temperature, primer hairpins, self-dimers, heterodimers, and individual and total ΔG was performed using PCR Primer Stats^3^ and IDT Oligo Analyzer Tools.^4^ The ΔG accepted for a dimer analysis was less than −6.5 kcal/mol.

The specificity of the amplicons was checked using the virtual nucleotide basic local alignment search tool (NCBI nucleotide BLAST)^5^ and by standard PCR with recombinant Taq DNA Polymerase (Thermo Scientific, Waltham, MA, United States), with the total DNA extracted from 50 mg of the frozen sample (DNeasy Plant Pro kit, Qiagen, Hilden, Germany) according to the protocol of the manufacturer to obtain a single band for each primer pair also in the genome, demonstrating its specificity and effectiveness. The efficiency of the primer sets was evaluated using the software QuantStudio 5 (QuantStudio^TM^ Design and Analysis Software version 1.4.0.0), by analyzing the threshold cycle (Ct)/fluorescence ratio at six independent points of PCR curves (Ramakers et al., 2003), obtaining the values between 95 and 100%. Five housekeeping genes [phospholipase A2 (PLA2), cyclophiling 2 (CYP2), elongation factor 1-alpha (Ef1α), ribosomal protein L13 (RPL13), and tubulin alpha (TUA)] from *Prunus sp.* were selected according to Klumb et al. (2019) and their primers were checked with each cDNA used in the quantitative PCR (qPCR). They were then measured using a Visual basic application for Excel (GeNorm) that automatically calculates the gene stability (Vandesomplete et al., 2002); the worst-scoring gene was discarded and the Normalization Factor (NF) was calculated using the three most stable constitutive genes.

Finally, the expression level of all the AQP genes was measured using the CFX Real-Time qPCR system (Bio-Rad, Hercules, CA, United States) from 2 μl of 1:10 diluted cDNA following the system instructions. Only the AQPs with a high expression and clear variation pattern between treatments were then selected. Final primers (forward and reverse sequences) used in this analysis and their efficiencies are shown in Table 1. They correspond to the four AQPs from the PIPs group, one PIP1 and three PIPs2 (XM 034348745.1, XM 034363911.1, XM 031370909.1, and XM 034366884.1 accessions in NCBI, respectively), and five from the TIPs group, two TIPs1, one TIP-type (TIP2), one TIP3, and one TIP4 (XM 034367674.1, XM 034373330.1, XM 034358950.1, XM 034359066.1, and XM 034344897.1 accessions in NCBI, respectively) (Table 1). Real-time PCR measurements were carried out in three independent RNA samples per treatment, and the Ct was determined three times. Expression levels were transformed from the quantification cycle values according to Die et al. (2010), using the primer efficiencies (Ramakers et al., 2003) and NF (GeNorm). Finally, normalized expression levels were rescaled and presented as a relative expression (r.e.) with respect to the control expression, which was assigned with a value of 100 before statistical analysis.

**TABLE 1:** Primers were used for the measurement of the selected *Prunus dulcis* aquaporins (AQPs) expression by real-time qPCR (RT-qPCR).

AQP group	Named	Forward primer (3′–5′)	Reverse primer (3′–5′)	NCBI accession	E (%)
PIPs1	PIP1.2	CAAGGACTACAAGGAGCC	ACAGGAAAAGGAAGGTGG	XM 034348745.1	97.90
PIPs2	PIP2.2	GATTCTCAGGAAAAGACTACC	GTGATGTACAAGAACAAGAGG	XM 034363911.1	100.20
	PIP2.4	GCTCGCAAGGTCTCGTTGAT	GAGTTGTAGTTGTGCTTCTGG	XM 031370909.1	99.35
	PIP2.3	CGTCATTGGCTACAAGTCC	GAATAGTCCAAAGGTCACAGC	XM 034366884.1	100.64
TIPs1	TIP1.1	CCAACTACCAGACTACCTC	CACGCTGCCTCTCTTCG	XM 034367674.1	103.04
	TIP1.2	CAGAGATGTGGAGAGCAG	ATGAGATGATTGAAGAGGTCAG	XM 034373330.1	104.06
TIPs3	TIP3.2	GCTCTGTTCTCGCACTTGG	GGTTACAGCAGGGTTGACG	XM 034359066.1	97.62
TIPs4	TIP4.1	CACTGGGTTTACTGGGTTG	AATGGGGAGATGAGTTGTTGG	XM 034344897.1	98.90
TIPs-type	TIP2.2	GTGAAGTTGGCTTTTGGTAGC	AAGGGTGGCAATGAACTCAGC	XM 034358950.1	101.76

**Constitutive genes**				**References**	

	CYP2	ACTCCAAAGCGTGTTAGAAAAGG	GTCTCTTCCACCATAACGATAGG	Tong et al., 2009	101.89
	RPL13	GCAGCGACTGAAGACATACAAG	GGTGGCATTAGCAAGTTCCTC	Tong et al., 2009	102.81
	TUA	TTCTCTCTACTCATTCCCTCCTTG	GATTGGTGTATGTTGGTCTCTCG	Tong et al., 2009	102.81
	PLA2	TCGCCGTCGTTATCTTCTCC	TACCGAATCCCAACAGAATTACAG	Tong et al., 2009	103.01
	EF1α	AATTGCCTTTGTTCCCATCTCTG	TGGGCTCCTTCTAATCTCCTTA	Xu et al., 2008	98.02

*Columns: AQP group (up) and constitutive genes (down), named (gene assigned names), forward primer sequence, reverse primer sequence, NCBI accession (up), and Reference (bibliographic references) for constitutive genes sequences (down), efficiency (E) in%.*

### Data Analysis

The data were subjected to the Kolmogorov–Smirnov test to check their normality. As the values followed a normal distribution, they were subjected to a simple ANOVA at a 95% confidence level, using the SPSS Release 18 software for Windows (SPSS, Inc., Chicago, IL, United States). The presented values are the means ± SEM. The significance levels for both analyses were expressed as: ^∗^*p* < 0.05, ^∗∗^*p* < 0.01, ^∗∗∗^*p* < 0.001, and different letters indicate significant differences (*p* < 0.05) as determined by Duncan’s multiple range and least significant difference tests (*n* = 4 for real-time qPCR (RT-qPCR) test and *n* = 8 for the rest of data).

## Results

### Plant Growth

**FIGURE 1:**
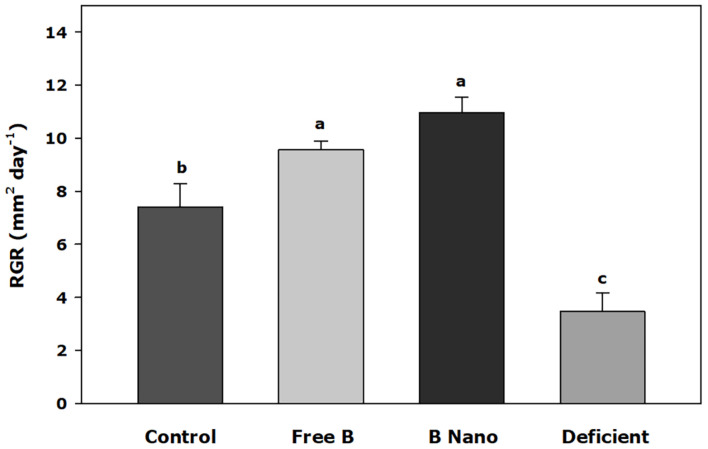
RGR of the youngest leaves 7 days after the foliar application of boron (B). B Nano, Nanoencapsulated B. Values are means ± SE (*n* = 8). Two independent experiments have been carried out. Columns with different letters differ significantly according to Duncan’s test (*p* < 0.05).

Figure 1 shows the relative growth of the meristematic leaves 7 days after the application of treatments. B application treatments showed a greater increase of the relative growth (RGR) of meristematic leaves than control treatments, whereas the B-deficient treatment obtained the lowest value, with significant differences in all other treatments. The highest values were found in plants treated with nanoencapsulated B *via* a foliar application, and no significant differences were found in plants treated with the free application of B.

### B Concentration in Different Plant Parts

Boron concentrations are shown in Table 2. After 2 h (S0) of B foliar application in the part of the plant, the results showed a significant increase in both free B and nanoencapsulated B, but the increase was much higher in nanoencapsulated B. B concentration in deficient plants was significantly lower than that in the control. In the part of the plants where B was not applied, no differences were found between deficient, free B, and nanoencapsulated B, with still significantly lower values than the control. The concentration of B at S0 in the meristematic leaves and roots was similar to that of the non-applied part of the plant, with similar and lower values in deficient, free B, and nanoencapsulated B treatments as compared to the control.

**TABLE 2:** Boron (B) concentration (μg g^–1^ DW) into almond tissues after the foliar application of B was measured at different times of collection (S0 and S1).

	Fully expanded leaves			Youngest leaves		Roots	
	S0		S1	S0	S1	S0	S1
Treatments	Applied	Non-applied					
Control	32.55 ± 2.14c	33.41 ± 5.24a	34.92 ± 2.33c	26.59 ± 3.06a	24.23 ± 0.21c	24.39 ± 2.18a	22.25 ± 2.64a
Free B	61.54 ± 4.58b	17.62 ± 2.24b	48.11 ± 1.14b	13.22 ± 1.89b	36.35 ± 1.76b	14.58 ± 1.09b	16.03 ± 0.96b
B Nano.	112.24 ± 8.15a	18.33 ± 1.20b	76.36 ± 3.48a	12.49 ± 1.32b	54.11 ± 2.23a	13.11 ± 1.37b	19.69 ± 1.27a
Deficient	19.03 ± 2.48d	20.63 ± 2.18b	13.27 ± 1.24c	12.77 ± 1.14b	11.91 ± 0.66d	12.55 ± 1.83b	11.82 ± 0.48c
*P-*value	***	**	***	*	***	*	**

Values are means ± SE (n = 8). Two independent experiments have been carried out. Levels of significance are represented by *p < 0.05; **p < 0.01; and ***p < 0.001. For each treatment, different letters show significant differences according to Duncan’s test at p < 0.05. B Nano., Nanoencapsulated B.

On the other hand, after 7 days (S1), the results showed significant differences in all the treatments and tissues. Plants treated with B showed significantly higher values than control plants in both fully expanded leaves and meristematic leaves. In both tissues, the nanoencapsulated B treatment produced the highest concentrations, with values that were double than those of the control. Nevertheless, the final B concentration in roots was significantly lower in the free B application treatment than in control plants, whereas in the nanoencapsulated B-treated roots, the levels reached were similar to those of control plants, without any significant differences between them.

### Foliar Absorption of B nanovesicles

**FIGURE 2:**
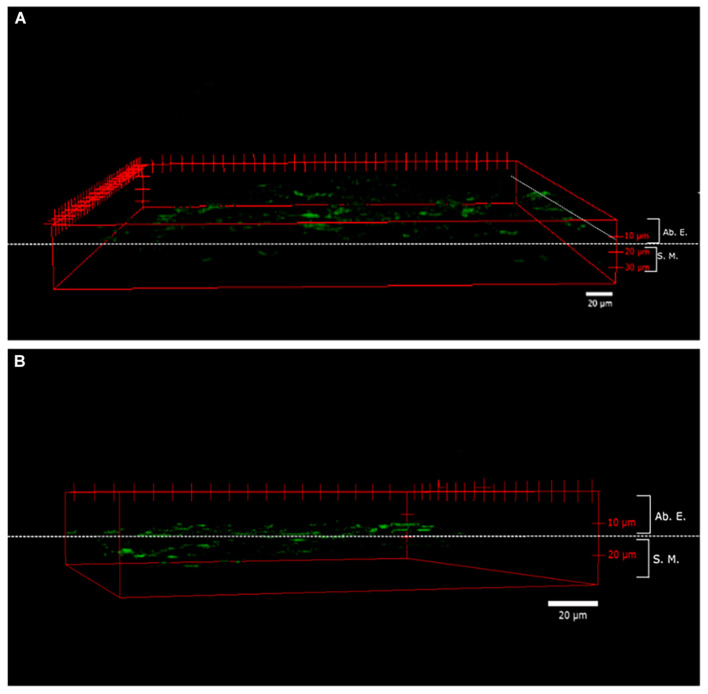
Penetrability of fluorescein disodium salt (FNA) in whole almond leaves. A three-dimensional (3D) reconstruction of the images obtained through a confocal laser scanning microscope (CLSM) of almond leaves from the abaxial epidermis to adaxial epidermis, 2 h after FNA- encapsulated foliar application: superficial expansion view **(A)** and the depth of nanovesicles view **(B)**. Ab. E., abaxial epidermis; S.M., spongy mesophyll.

Figure 2 shows the images of the area and depth of B entry into the layers of leaf tissues taken by CLSM 2 h after a foliar application. The observed fluorescence area was homogeneous along the entire surface, indicating that the nanovesicles were mainly entered through the cuticle to reach the abaxial epidermis (Figure 2A). The fluorescence was found to be mostly between 10 and 20 μm from the cuticle (0 μm). In addition, the depth of penetration observed at this time is shown, revealing that, although most of the fluorescence was found in the epidermis, other fluorescence areas were observed for entering the next layer, that is, the spongy mesophyll (Figure 2B).

### B Concentration in the Cell Wall

**FIGURE 3:**
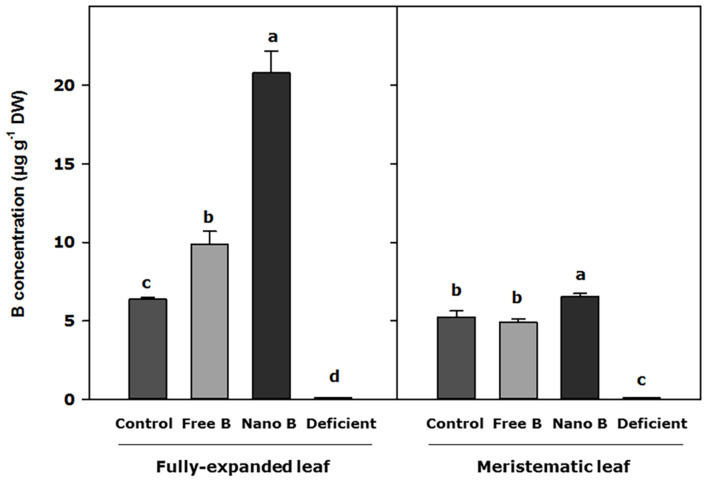
B concentration in the cell wall of almond leaves 7 days after the foliar application of B. B Nano, Nanoencapsulated B. Values are means ± SE (*n* = 8). Two independent experiments have been carried out. Columns with different letters differ significantly according to Duncan’s test (*p* = 0.05), separately fully expanded leaf and meristematic leaf.

Boron concentration in the cell wall increased in fully expanded leaves 7 days after B application, with significant differences observed between treatments. In this regard, nanoencapsulated B produced the highest values of B assimilation into the cell wall (3 times higher than the control), and free B increased B concentration in plant cells 1.4 times as compared to control plants (Figure 3). However, the assimilation of B into the cell wall of meristematic leaves only showed significant differences with respect to control plants when B was applied as nanoencapsulated B, observing an enhanced B concentration in meristematic leaves 7 days after the nanoencapsulated B application (Figure 3).

### Water Relations and Root Hydraulic Conductance

**FIGURE 4:**
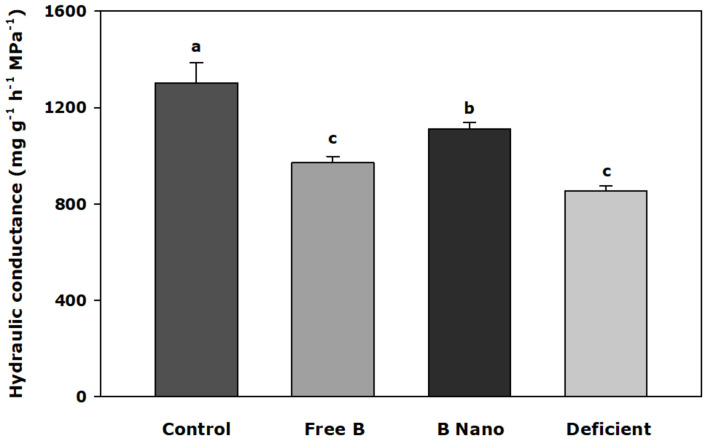
Hydraulic conductance of the almond root 7 days after the foliar application of B. B Nano, Nanoencapsulated B. Values are means ± SE (*n* = 8). Two independent experiments have been carried out. Columns with different letters differ significantly according to Duncan’s test (*p* = 0.05).

Figure 4 shows the values of root hydraulic conductance (*L*_*h*_) 7 days after B application. The results indicated significant differences between treatments (*p* < 0.001), with the highest values obtained by control plants. B treatments showed a decrease in root conductance, which is higher in the case of deficient plants and also larger in the case of plants treated with nanoencapsulated B.

The water relations of fully expanded leaves and meristematic leaves are shown in Table 3. The results for both parts of the plants were similar, with significant changes only observed in water potential (Ψ_*w*_). For this parameter, 2 h after treatment application (S0), the highest values were observed in control plants, and no differences were found between the other treated or non-treated fully expanded leaves. However, 7 days after B treatment (S1), Ψ_*w*_ increased in B-treated plants by a foliar application, until the values similar to those of control plants were obtained in both fully expanded and meristematic leaves, where no significant differences were found depending on the method of B application. The lowest values were observed in B-deficient plants.

**TABLE 3:** Leaf water relations (water potential Ψ_*w*_, osmotic potential Ψ_π_, and turgor Ψ_*t*_ all expressed in MPa) of fully expanded and the youngest leaves of almond after the foliar application of B measured at different times of collection (S0 and S1).

	Ψ_*w*_				Ψπ				Ψt			
	S0		S1		S0		S1		S0		S1	
Treatments	Applied	Not applied	Expanded	Youngest	Applied	Not applied	Expanded	Youngest	Applied	Not applied	Expanded	Youngest
Control	−0.38 ± 0.03a	−0.39 ± 0.01a	−0.39 ± 0.02a	−0.17 ± 0.01a	−2.69 ± 0.10a	−2.72 ± 0.12a	−2.81 ± 0.07a	−1.43 ± 0.05a	2.31 ± 0.07a	2.33 ± 0.08a	2.42 ± 0.11a	1.26 ± 0.15a
Free B	−0.22 ± 0.05b	−0.28 ± 0.03b	−0.34 ± 0.04a	−0.18 ± 0.02a	−2.67 ± 0.26a	−2.80 ± 0.19a	−2.90 ± 0.13a	−1.41 ± 0.12a	2.45 ± 0.11a	2.52 ± 0.12a	2.56 ± 0.18a	1.23 ± 0.11a
B Nano.	−0.23 ± 0.04b	−0.28 ± 0.04b	−0.40 ± 0.01a	−0.19 ± 0.01a	−2.75 ± 0.20a	−2.82 ± 0.19a	−2.83 ± 0.16a	−1.42 ± 0.08a	2.51 ± 0.15a	2.54 ± 0.20a	2.43 ± 0.10a	1.23 ± 0.14a
Deficient	−0.27 ± 0.02b	−0.27 ± 0.03b	−0.26 ± 0.01b	−0.12 ± 0.01b	−2.86 ± 0.23a	−2.90 ± 0.26a	−2.85 ± 0.17a	−1.47 ± 0.06a	2.59 ± 0.16a	2.63 ± 0.18a	2.59 ± 0.23a	1.35 ± 0.10a
	***	*	***	*	ns	ns	ns	ns	ns	ns	ns	ns

Values are means ± SE (n = 8). Two independent experiments have been carried out. Levels of significance are represented by *p < 0.05; **p < 0.01; and ***p < 0.001 or ns. Different letters indicate significant differences among treatments according to Duncan’s test (p < 0.05). B Nano, B nanoencapsulated.

### Gas Exchange Parameters

The effects of B application on gas exchange in fully expanded leaves are summarized in Table 4. Transpiration, CO_2_ assimilation rate, and WUE did not change between the leaves where B was applied or not and was applied to fully expand leaves (S0) after 2 h although significant differences were found between control plants and the rest of the treatments, with higher values observed in control plants. However, parameters, such as stomata conductance and internal CO_2_ (CI), showed significant differences between treatments and B-applied and non-B-applied leaves. In this regard, control plants obtained the highest values of stomatal conductance, and the lowest were found in leaves after B application for both free and nanoencapsulated B treatments. Conversely, the lowest CI values were found in the control treatment, and all the treatments obtained higher values with respect to the control. A reduction of both parameters (stomatal conductance and CI) was observed by B application on the leaves where it was supposed to be applied with respect to the ones where B was not applied.

**TABLE 4:** Gas exchange parameters such as transpiration, stomatal conductance, internal carbon dioxide (CO_2_, mmol m^–2^ s^–1^), and assimilation (μmol m^–2^ s^–1^) and water use efficiency (WUE, in μmol CO_2_ mmol H_2_O^–1^) of the fully expanded and youngest leaves of almond after the foliar application of B measured at different times of collection (S0 and S1).

A	Transpiration				Stomatal conductance				Assimilation rate			
	S0		S1		S0		S1		S0		S1	
Treatments	Applied	Not applied	Expanded	Youngest	Applied	Not applied	Expanded	Youngest	Applied	Not applied	Expanded	Youngest
Control	2.56 ± 0.34a	2.76 ± 0.14a	3.82 ± 0.15a	3.50 ± 0.15a	515.60 ± 29.72a	505.60 ± 35.72a	608.8 ± 34.03a	382.80 ± 21.44b	8.57 ± 0.33a	8.67 ± 0.33a	10.91 ± 0.44b	8.29 ± 0.24b
Free B	1.59 ± 0.09b	1.93 ± 0.07b	3.08 ± 0.14b	3.42 ± 0.17a	290.40 ± 14.53c	322.41 ± 27.39b	514.62 ± 15.47b	456.61 ± 14.66a	4.74 ± 0.21b	4.38 ± 0.12b	11.22 ± 0.43b	9.09 ± 0.37b
B Nano.	1.72 ± 0.14b	1.89 ± 0.09b	3.74 ± 0.10a	3.64 ± 0.14a	307.20 ± 25.78c	333.68 ± 19.55b	586.24 ± 25.03a	464.33 ± 12.22a	4.88 ± 0.16b	4.23 ± 0.10b	13.80 ± 0.86a	11.19 ± 0.43b
Deficient	1.79 ± 0.12b	1.93 ± 0.11b	1.97 ± 0.07c	1.57 ± 0.12b	367.38 ± 23.71b	352.38 ± 15.01b	334.11 ± 16.76c	294.53 ± 12.1c	4.32 ± 0.48b	4.19 ± 0.76b	4.79 ± 0.74c	3.09 ± 0.27c
*P-*value	***	***	**	**	**	**	*	**	*	*	**	*

B	Internal CO_2_				Stomatal conductance			
	S0		S1		S0		S1	
Treatments	Applied	Not applied	Expanded	Youngest	Applied	Not applied	Expanded	Youngest
Control	434.62 ± 15.66c	411.62 ± 28.29c	434.41 ± 23.998b	346.2 ± 17.24b	3.44 ± 0.16a	3.15 ± 0.12a	2.96 ± 0.10b	2.38 ± 0.10b
Free B	496.44 ± 12.12b	535.23 ± 31.58ab	393.65 ± 21.55b	363.8 ± 10.20b	3.03 ± 0.11b	2.27 ± 0.15c	3.68 ± 0.28a	2.68 ± 0.12b
B Nano.	488.45 ± 13.31b	540.02 ± 20.86ab	425.94 ± 34.58b	338.01 ± 15.73b	2.91 ± 0.27b	2.24 ± 0.12c	3.69 ± 0.19a	3.09 ± 0.14a
Deficient	583.02 ± 33.12a	595.43 ± 47.82a	535 ± 46.84a	512.32 ± 21.94a	2.41 ± 0.18b	2.19 ± 0.11c	1.87 ± 0.12c	1.95 ± 0.19c
*P-*value	**	**	*	**	*	*	***	***

Levels of significance are represented by * p < 0.05; ** p < 0.01; and *** p < 0.001. Different letters indicate significant differences among treatments according to Duncan’s test (p < 0.05). Values are means ± SE (n = 8). Two independent experiments have been carried out.

After 7 days (S1), all the values changed depending on the treatment. Indeed, the results showed significant differences in all the measured parameters, with increases in the values after B application treatments as compared to deficient plants. The only exception was CI, in which B-treated plants did not produce any changes as compared to control plants. In fully expanded leaves, control and nanoencapsulated B treatments showed the highest values in transpiration and stomatal conductance, with the lowest value obtained by deficient plants. Regarding the assimilation rate of the expanded leaves, the nanoencapsulated B treatment had an increased rate, reaching values that were higher than those of the rest of the treatments 7 days after B application. As previously mentioned, CI was only significantly different in the B-deficient plant, which shows the highest values. In addition, WUE values were higher than control plants after B treatments, obtaining the highest results in this parameter, while the deficient plants again show the lowest values. For the meristematic leaves after 7 days from B application treatments, the results showed an improvement in all the parameters, and significant differences between treatments were observed. For transpiration, stomatal conductance, assimilation rate, and WUE, the lowest values were shown by deficient plants. In addition, there were no differences between B treatments and control plants, with an exception of the stomatal conductance parameter of which control plants exhibited lower values than B-treated plants. As for WUE, the highest value was found with the application of nanoencapsulated B, while control plants and free B-treated plants showed similar results. In this case, it was again B-deficient plants the ones obtaining the lowest values for all these parameters (Table 4).

### The Expression of Aquaporins in Fully Expanded Leaves at the Initial Time (S0)

**FIGURE 5:**
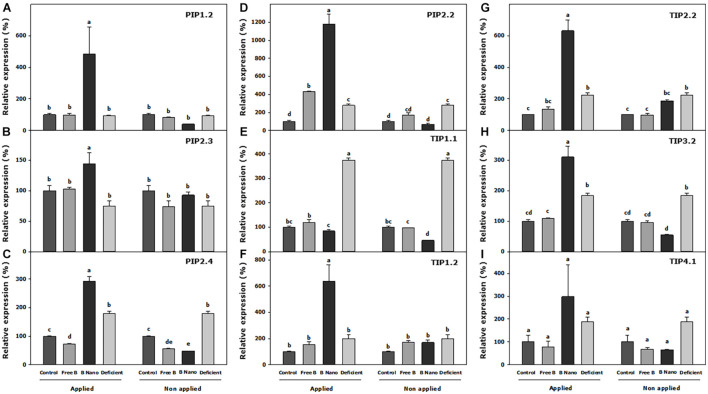
Expression analysis of **(A)** PIP1.2, **(B)** PIP2.3, **(C)** PIP2.4, **(D)** PIP2.2, **(E)** TIP1.1, **(F)** TIP1.2, **(G)** TIP2.2, **(H)** TIP3.2, and **(I)** TIP4.1 *Prunus dulcis* aquaporins (AQPs) at S0. Statistical analysis was performed including all B-applied and non-B-applied leaves, separating each AQP. Normalized expression levels were rescaled and presented as a relative expression (r.e.) with respect to the control. B Nano, Nanoencapsulated B. The values are the means ± SE of four individual analyses. Columns with different letters differ significantly according to Duncan’s test (*p* = 0.05).

Figure 5 shows the results of the expression of AQPs in fully expanded leaves at S0. Thus, B deficiency produces a slight increase in the expression of AQPs, which is significant in PIP2.2, PIP2.4, TIP2.2, TIP3.2 and is especially striking in the case of TIP1.1, where the expression level of the control increased 3.7 times. With B application, the most remarkable result obtained was that all the analyzed AQPs showed a strong and rapid increase in expression in the leaves where nanoencapsulated B was applied, as compared to all the other treatments. The most important increase was observed in PIP2.2, with around 11-times more expression than the control. In the free B foliar application treatment, in general, there were no changes in the expression of AQP genes. The only exceptions were PIP2.2 and TIP1.1, which increased their expression in the leaves where B was applied (2.5 and 1.2 times, respectively) as compared to the leaves from the same B treatment where B was not applied.

### Expression of Aquaporin Genes in Fully Expanded and Meristematic Leaves 7 Days After B Application (S1)

**FIGURE 6:**
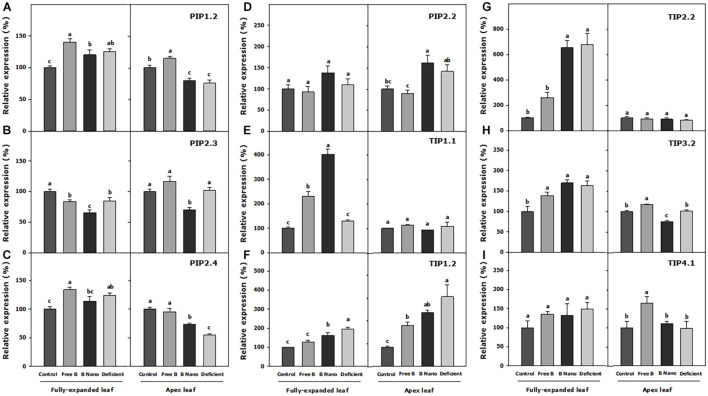
Expression analysis of **(A)** PIP1.2, **(B)** PIP2.3, **(C)** PIP2.4, **(D)** PIP2.2, **(E)** TIP1.1, **(F)** TIP1.2, **(G)** TIP2.2, **(H)** TIP3.2, and **(I)** TIP4.1 *P. dulcis* AQPs at S1. Statistical analysis was performed for separating different tissues, fully expanded, and the youngest leaves. Normalized expression levels were rescaled and presented as r.e. with respect to the control. B Nano, Nanoencapsulated B. The values are the means ± SE of four individual analyses. Columns with different letters differ significantly according to Duncan’s test (*p* < 0.05).

Figure 6 shows the expression of AQP genes 7 days from the application of B treatments. With respect to the expanded leaves, all the treatments produce an increase or the maintenance of the expression of AQPs in fully expanded leaves at S1 as compared to the control. A remarkable exception was *PIP2.3*, the only AQP with a decreased expression in the B-deficient treatment as well as in the applied B treatments as compared to the control. Both B deficiency and B application treatments obtained similar values in the expression of PIPs at this time, while the most notable differences were found in the TIP subgroup. In this regard, B application increased *TIP1.1* expression notably with respect to control and B-deficient treatments, with this value being greater when nanoencapsulated B was applied. *TIP1.2* and *TIP2.2* expressions increased in the nanoencapsulated B treatment but not in the free B treatment, and the expression of *TIP3.2* increased in both B treatments. In addition, the B-deficient treatment enhanced the expression of all these AQPs in a similar manner as nanoencapsulated B. *TIP4.1*, on its part, was not affected by any treatment.

In the meristematic leaves, the expression of *TIP1.1* and *TIP2.2* did not change with any of the treatments, while *TIP1.2* had an increased expression in all the treatments as compared to the control, reaching the highest expression values in all the treatments. The free B foliar application either increased or did not modify the expression of the AQP genes in the meristematic leaves at S1. More specifically, the expression of *PIP1.2*, *PIP2.4*, *TIP3.2*, and *TIP4.1* increased, and notably, this treatment was the only one, which changed the expression pattern of *TIP4.1*, causing an increase of 63% as compared to the control. The B-deficient treatment and the foliar application of nanoencapsulated B had almost similar patterns of expression for all the AQPs analyzed in the meristematic leaves, with the exception of *PIP2.3* and *TIP3.2*, which were downregulated by the nanoencapsulated B treatment, and *PIP2.4*, which was upregulated although it did not obtain the values as high as in the treatment with free B.

## Discussion

The application of fertilizers to the soil has been reported to have low efficiency in almond trees compared to foliar sprays (Arrobas et al., 2019), which is proved to be a strategy for improving the nutritional status of trees. Indeed, studies on the application of foliar fertilization revealed that the sprays applied at post-harvest could enhance flowering the following spring (Saa et al., 2017) providing nitrogen and B. Among micronutrients, B deficiency is the most important disorder affecting tree crop growth and yield, as reported from the other important tree crop species grown in the Mediterranean area, such as olive (Rodrigues et al., 2011), chestnut (Arrobas et al., 2018), and pistachio (Güneş et al., 2010). Indeed, B fertilization of *Prunus* trees has long been reported to increase growth (Wójcik, 1999) as it is necessary for tree fertility and yield (Nyomora et al., 2000). In our results, B-deficient trees were highly decreased in new growth areas. However, when extra B was applied *via* a foliar application, a significant increase was observed with no significant differences between free B and nanoencapsulated B.

According to B concentration in almond tree tissues, it has been reported that, when B is taken by the roots, the transport of B to the aerial part is being driven by the transpiration stream although it tends to accumulate in older leaves (Brown et al., 2002) if B is not progressively supplied. In our experiments, the leaf application of B did not allow its movement through the transpiration stream. Therefore, B remained mainly in the tissue where it was applied when it was supplied in its free form, as observed in the results shown in Table 2. However, when B was supplied in a nanoencapsulated form, apart from the appearance of higher concentrations within the tissue where it was applied, its mobility was much higher than in its free form. Therefore, a high increase of B concentrations in the youngest leaves and even in the roots, suggests that the nanoencapsulation promoted the movement of B within the plant, making unnecessary its movement with the transpiration stream. Although previous studies such as Brown and Hu (1996) and Nyomora et al. (2000) indicated that *Prunus* spp. has high phloem mobility because these trees are sorbitol-rich species and this sugar helps in B phloem mobility. Our results showed that nanoencapsulated B had more penetration and mobility when B was applied in a non-encapsulated form. In this way, the results in Figure 2 show that nanocapsules containing FNA were able to penetrate leaf tissues in 2 h, demonstrating that this could favor absorption through the epidermis as suggested earlier (Rios et al., 2020). However, they can penetrate and reach other tissues, thereby allowing the transport of B toward other organs rather than those where B was applied. In this way, the encapsulated foliar application allowed B to be transported *via* phloem or through the symplastic pathway *via* transporters.

If we consider B localization within the tissues of the plant, it has been widely reported that around 90% of B remains in the cell walls (Läuchli, 2002) as it is identified as an essential micronutrient for the stabilities of cell wall constituents (Camacho-Cristóbal et al., 2008), and its transformation during growth (Brown et al., 2002). Indeed, the need for B has been linked to rhamnogalacturonan II (RGII) content (Reid et al., 2004) as a reduction of pectins or precursors in the cell wall was observed in B-deficient plants (Camacho-Cristóbal et al., 2008). The mechanism of regulation has been investigated through the synthesis of RGII, which requires B (Funakawa and Miwa, 2015). Our results point to the same direction, as B application increases after 7 days into the cell wall of fully expanded leaves, and nanoencapsulated B showed an increment in the cell wall of the youngest leaves too, pointing to a better efficiency in reaching all plant tissues better than the applied free B. Apart from the essential function on the cell wall, several works have suggested that B plays a structural and regulatory role inside the plasma membrane (Bastías et al., 2010). This study suggests that an increase of AQP functionality due to the presence of B is a key point of the results that should be linked to cell wall stability, which is needed for plant growth.

Interestingly, it has been reported that B toxicity leads to a rapid decrease in water uptake and transport that are driven by stomatal opening in Arabidopsis plants, which could be a mechanism for limiting the transport of excess B from the root to the shoot (Macho-Rivero et al., 2017, 2018) although B deficiency produces similar effects (Huang et al., 2005). These results could point to a fine adjustment of whole plant water relations to restrict B accumulation in plant tissues in response to B toxicity, or to restore B accumulation in response to B deficiency (Barzana et al., 2020). Our results obtained from the root hydraulic conductance measurements suggest a relationship with B concentration in tissues and this parameter is to be decreased as in B-deficient roots and in plants treated with free B where B concentration was low. However, in plants treated with nanoencapsulated B, with a concentration similar to the control, the root hydraulic conductance value was only slightly reduced from that found with the control.

The results of gas exchange parameters revealed that they were related to B concentration in leaves. Thus, reductions in leaf net CO_2_ assimilation rate, transpiration, and stomatal conductance in the leaves of both rootstocks were observed in deficient plants, which were restored when B was supplied. A higher restoration in leaf gas exchange parameters occurred when B was nanoencapsulated. This effect has been reported previously, thereby proposing that a direct relationship between the reduction in a leaf gas exchange in response to B deficiency is due to a decrease in B in the leaf (Quamruzzaman et al., 2017). However, the associated mechanism has not been elucidated. The restoration of photosynthetic capacity, CI, stomatal conductance, and transpiration, when both B treatments were applied, suggests an interactive effect on membrane water and CO_2_ transport, which involves AQPs. In this way, an increase in WUE was apparently due to an increase in both stomatal conductance and photosynthetic capacity with the regulation of related AQPs. Indeed, *L*_*h*_ was also restored in the same manner, pointing to the involvement of water-transporting AQPs.

In the conditions of changing transpiration demands, water uptake must balance water consumption (Bastías et al., 2004). In our experiments, the non-alteration of water potential, osmotic potential, and turgor potential in any of the leaves (B-applied, non-applied, and meristematic) suggests that almond trees are osmotically adjusted. Thus, water uptake could have balanced water content, granting the AQPs a key role in determining the plant water status.

According to AQP expression analysis in our almond trees, the only reduction in the expression of AQP *PIP2.3* in B-deficient plants revealed that it could be the AQP that mostly influenced water transport, resulting in the reduction of stomatal conductance, transpiration, and *L*_*h*_ in the leaves of those plants. Interestingly, in B application treatments, the regulation based on *PIP2.3* did not seem to be the reason behind the recovery in these parameters. In these cases, a strong correlation was not found with the increases of PIPs at S1. Nevertheless, the AQPS could be regulated at different times so that the effects of their expressions could then only have an effect days after when the proteins are synthesized and incorporated into the different membranes (Bárzana et al., 2014). In that way, an increase in the expression of all the AQPs in the leaves where nanoencapsulated B was applied at S0, especially *PIP2.2*, is noted. The structure of PIPs is highly preserved, and their function in water transport has been reported to be indispensable for plants grown in soils (Zardoya, 2005; Anderberg et al., 2012). All the PIPs have the same asparagine–proline–alanine (NPA) motifs and ar/R selectivity filter residues (F, H, T, and R in H2, H5, LE1, and LE2, respectively) predicting a high selectivity for water transport (Sui et al., 2001). The increased PIP expression observed correctly after the application of B (S0) could then be related to a striking recovery of the gas exchange, *L*_*h*_, and WUE parameters days after, as observed in both fully expanded and meristematic leaves at S1. However, in the non-encapsulated B application treatment, only *PIP2.2* and *TIP1.1* were initially promoted and the recovery in the physiological parameters was equally significant, PIP2.2 being the common factor implicated in such a recovery regardless of how B is applied. All of these suggest that in *P. dulcis* L. trees, the PIP2.2 water-transporting AQPs play a fundamental role in the recovery from B stress. In the nanoencapsulated B treatment, the possible function of *PIP2.2* seems to be complemented by an increase in the expression of the rest of the AQPs.

It has been reported that under an adequate B supply, B uptake and transport through the plant are passively driven by the transpiration stream, and B entry to cells is associated with a simple diffusion through a lipid bilayer, or facilitated diffusion through channels (Brown et al., 2002). However, under B deficiency, an active transport system *via* B transporters has been suggested (Takano et al., 2002; Nakagawa et al., 2007). PIPs that have been shown to transport boric acid, urea, and arsenic and their sequence homology analysis result in the prediction of this transport due to the function of orthologous genes (Perez Di Giorgio et al., 2014). According to this hypothesis, CmPIPs1 has been reported to be able to transport boric acid (Lopez-Zaplana et al., 2020) as its orthologous ZmPIP1;1 and ZmPIP1;5 (Dordas et al., 2000; Gaspar et al., 2003). The fundamental selectivity filters of PIP1.2, PIP2.3, PIP2.4, and PIP 2.2 are similar to AQPs associated with B transport. This PIP positively responded to the application of nanoencapsulated B, and could therefore indicate the need for B transport as a high concentration reached the cells in a short time. In this way, it can be observed that in the S1 experiments, the expression of the latter AQPs decreased to normal values. Therefore, in those plants, there was no need to alter the expression of PIP AQPs to adjust B movement no longer. In contrast, the enhanced expression of *PIP1.2* and *PIP2.4* was observed in the meristematic tissues of the plants after the application of free B could point to B transport needs, accompanied with enhanced transpiration and stomatal conductance in these tissues, which resulted in the same values as nanoencapsulated B plants.

On the other hand, it has been suggested that B could be involved in the structure of the membranes named as “lipid rafts,” as it could be bound to glucosilfosfatil-inositol proteins (Brown et al., 2002). The “lipid rafts” are characterized by high concentrations of glycolipids and glycoproteins, providing a significant number of B complexing sites, but also a large number of AQPs have been described (Yepes-Molina et al., 2020), which confer high stability to the plasma membrane. Therefore, in addition to all the molecules that could be able to link with B, an increase in the expression of the observed PIP1 and PIPs2 AQPs in our almond tress after the treatment with encapsulated B could indicate that B plays a function in the stability, integrity, and water transport function of “membrane rafts.” Furthermore, glycosylinositol phosphoryl ceramides (GIPCs), the major sphingolipids in lipid rafts, were reported to form a GIPC–B–RGII complex (Borner et al., 2005) and could be the molecular wall-membrane attachment sites (Voxeur and Fry, 2014). This could provide further evidence of the role of structural B in an increase in the expression of PIP AQPs, which increase water transport in cells.

In the case of TIPs, based on the ar/R filter, they were classified into four groups according to homology, as described by Wallace and Roberts (2004). In our case, Group I was formed by TIP1.1, Group IIa by TIP2.2, Group IIb was constituted by TIP3.2 and TIP4.1, whereas TIP1.2 was classified to another non-defined TIP subclass, based on the variations in its amino acid sequence. Except for TIP1.2, the TIPs have conserved NPA motifs and the characteristic ar/R filter of each subgroup (Group I, H-I-A-V, Group IIa, H-I-G-R, and Group IIb, H-I-A-R). In general, TIPs seem to develop the capacity to transport nitrogenous compounds [urea and ammonia (NH_3_)] (Gerbeau et al., 1999). In addition, TIPs1 has been described as the most important tonoplast AQP related to transcellular water movement (Maurel et al., 2008). However, *Zea mays* L. AQPs, ZmTIP1;1 and ZmTIP1;2 were able to transport boric acid in addition to NH_3_, urea, and water (Bárzana et al., 2014), and the selectivity filter of TIP1.1 (in *P. dulcis* H, I, A, and V) was related with N and B transport functions based on a phylogenetic framework and homologous analysis (Lopez-Zaplana et al., 2020). Reduced transpiration can lead to striking B deficiency due to a decreased absorption and mobility of this compound, suggesting that B requirements should be guaranteed by the B AQPs that can transport it, including TIPs1 (Bárzana et al., 2014). In this regard, a strong increase in *TIP1.1* in B-deficient plants at S0 points to a fundamental role in the remobilization of B from vacuoles in *P. dulcis* trees. Such an increase was much less pronounced in B application treatments and only affected the leaves where B was applied as compared to non-applied tissues despite its values being much lower than those found in deficient plants. In this case, TIP1.1 could work in B detoxification or accumulation of excess of B into the vacuole in the tissues where B enters in high amounts. Nonetheless, an increase in *TIP1.1* was notable in B application treatments at S1 in fully expanded leaves, confirming that TIP1.1 could be a function of B transport into and out of vacuoles. However, the most important function could be the remobilization of this compound from vacuoles to cope up with B requirements and the recovery of the physiological parameters, as shown in B-treated plants.

Interestingly, at S0, the expression of *TIP1.2*, *TIP2.2*, *TIP3.2*, and *TIP4.1* strongly increased in the nanoencapsulated B treatment but not in the free B treatment. This could be due to a high amount of B that rapidly reached the cells and could also be related to the vacuole storage of nutrients. In this way, the expression of *TIP1.2*, *TIP2.2*, and *TIP3.2* remained significantly higher in S1 in the fully expanded leaves as compared to controls, but the deficient plants also showed a significantly higher expression. In addition, it should be noted that the regulation of the TIPs in the free B-treated plants was slightly different. Meanwhile, *TIP1.1* and *TIP3.2* showed the same pattern as the nanoencapsulated B treatment in fully expanded leaves, there was a notable increase in *PIP1.2*, *PIP2.4*, *TIP3.2*, and *TIP4.1* expression in meristematic leaves. The phylogenetic analysis of TIP3.2 pointed to its role in N nutrition (Jahn et al., 2004), and in the case of TIP4.1, a specific histidine residue in loop C allowed the transport of NH_3_ (Kirscht et al., 2016). It is notable that these two AQPs only showed increases in their expression in this specific free B treatment and the meristematic tissue. Therefore, a modification in the plant metabolism due to the manner of B application could alter the nutritional balance in meristematic leaves. Thus, plants from the free B application treatment should more strongly adjust the transport of water and nutrients in meristematic leaves than plants treated with nanoencapsulated B.

Finally, it should be noted that the highest expression of *TIP1.2* was observed in meristematic tissues. While this AQP has been included as a TIP AQP, its specific amino acid residues are very different from all others, and no information about its possible specific functions has been described to date. Therefore, its involvement in the development of growing meristematic tissues should be further investigated.

## Conclusion

The interest in nanoparticles as nutrient carriers has increased due to their consideration as smart technology. In our work, the obtained results with nanoencapsulated B pointed to very high efficiency as compared to the free B application. The efficiency was proven to come from the effectiveness of vesicles to penetrate the leaf and reach the cell wall. The connection between the cell wall and the plasma membrane was also pointed out as a key site for further research, in relation to raft domains as it must be the interaction that triggers the regulation of AQP expression for allowing B transport and mobilization within the cell. All of these indicate a very fine regulation of B into the cells, depending on the amount of B and the speed with which it reaches the cells. Furthermore, a large number of AQPs responded to our treatments, and they are not only coped up with B owing to no change in the turgor but also granted special importance to PIP2.2 for water movement and TIP1.1 for B remobilization. In this regard, the fact that B could interact with the vesicle components sending membrane signaling could be explored.

## Data Availability Statement

The original contributions presented in the study are included in the article/Supplementary Material, further inquiries can be directed to the corresponding author/s.

## Author contributions

JR: conceptualization, methodology, formal analysis, investigation, and writing—original draft. AL-Z: investigation, writing—original draft, visualization, and resources. GB: data curation, writing—original draft, and writing—review and editing. AM-A: investigation and resources. MC: conceptualization, supervision, writing—review and editing, funding acquisition, and project administration. All authors contributed to the article and approved the submitted version.

## Conflict of Interest

The authors declare that the research was conducted in the absence of any commercial or financial relationships that could be construed as a potential conflict of interest.

## Publisher’s Note

All claims expressed in this article are solely those of the authors and do not necessarily represent those of their affiliated organizations, or those of the publisher, the editors and the reviewers. Any product that may be evaluated in this article, or claim that may be made by its manufacturer, is not guaranteed or endorsed by the publisher.

## Footnotes

1
https://www.ebi.ac.uk/Tools/msa/clustalo/

2
https://itol.embl.de/

3
https://www.bioinformatics.org/sms2/pcr_primer_st

4
https://eu.idtdna.com/calc/analyzer/

5
https://blast.ncbi.nlm.nih.gov/Blast.cgi
