# Supplementary material for: Foliar Application of Boron Nanoencapsulated in Almond Trees Allows B Movement Within Tree and Implements Water Uptake and Transport Involving Aquaporins
Source: Front Plant Sci. 2021 Nov 17;12:752648. doi: 10.3389/fpls.2021.752648 (PMC8636056; doi:10.3389/fpls.2021.752648)
Supplement: Supplementary file 1 [file Data_Sheet_1.docx]

**Foliar application of Boron nanobiofertilizers in almond trees allow B movement within tree and implements water uptake and transport involving aquaporins.**

Rios JJ, Lopez-Zaplana A, Bárzana G, Martinez-Alonso A., Carvajal M*

**Supplementary Figure 1**. Phylogenetic analysis of AQP family of Prunus sp. based in DNA sequences obtained in NCBI database. MUSLE was used to align the protein sequences and the NJ method (with 1000 bootstrap replications) to build the tree, all with MEGA X. Phylogenetic tree design has been done with the online tool “Interactive Tree Of Life” (iTOL; <https://itol.embl.de/>).


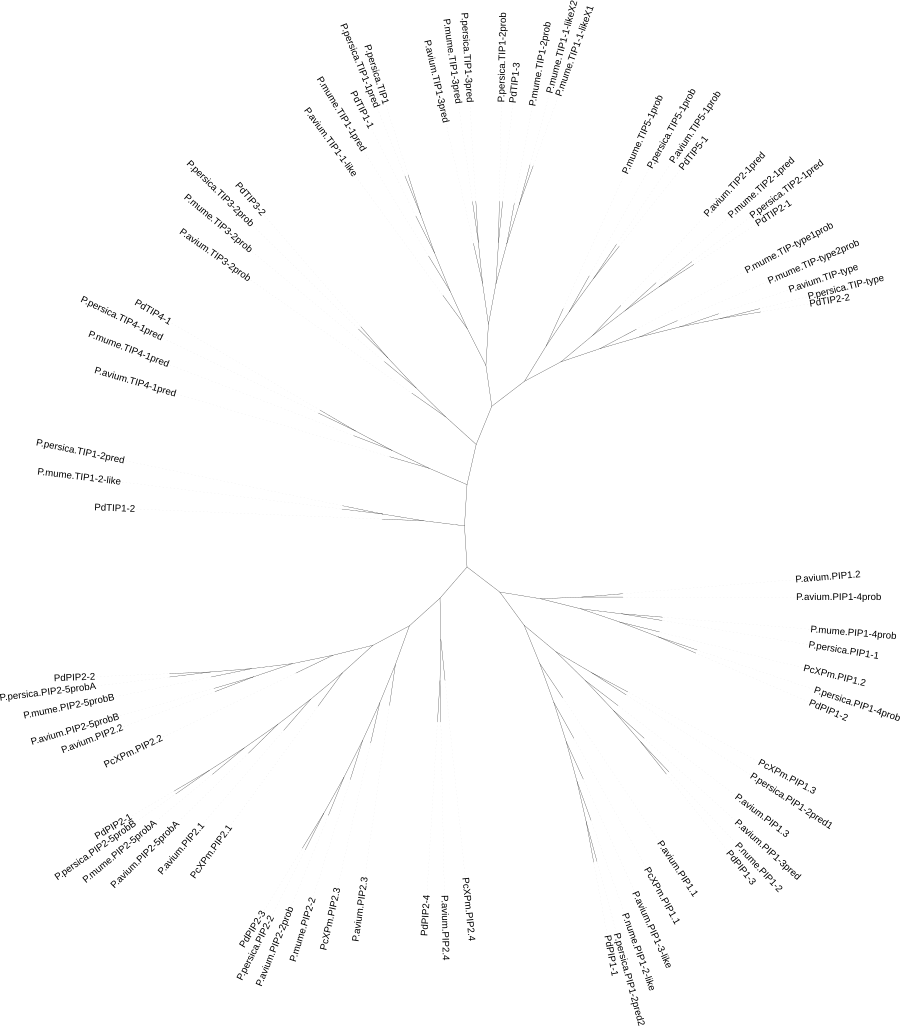


Shortening code: P: Prunus, Pd: *Prunus dulcis*, PIP: Plasma membrane intrinsic proteins, TIP: Tonoplast intrinsic proteins, prob: probable, pred: predicted, X: transcript variant. Names are assigned to our better understanding, the accession numbers in NCBI of sequences used are in supplementary table 1.

**Supplementary Table 1.** Gas exchange parameters transpiration, stomatal conductance, CO_2_ assimilation (µmol m^-2^ s^-1^) of almond full expanded leaves after foliar application of Control and after application of the encapsulation system. The different letters indicate significant differences among treatments according to Duncan’s test (P < 0.05). Values are means ± SE (n=8).

| Treatments | Transpiration | Stomatal conductance | Assimilation rate |
| --- | --- | --- | --- |
| Control | 2.84 ±0.37 | 543.50±46.84 | 9.21±0.21 |
| Nanoencapsulation system | 2.65 ±0.44 | 538.47±39.25 | 8.87±0.47 |

**Supplementary Table 2.** Names assigned to the PIP (left) and TIP (right) sequences of Prunus sp. present in NCBI database and its accession numbers. Columns: Assigned name, NCBI accession.

| **Assigned name** | **NCBI accesion** |  | **Assigned name** | **NCBI accesion** |
| --- | --- | --- | --- | --- |
| P.nume.PIP1-2 | XM_008240694.2 |  | P.mume.TIP1-1pred | XM_008243157.1 |
| P.nume.PIP1-2-like | XM_008234665.2 |  | P.mume.TIP1-1-likeX2 | XM_008224671.1 |
| P.mume.PIP1-4prob | XM_008235506.2 |  | P.mume.TIP1-1-likeX1 | XM_008224664.1 |
| P.mume.PIP2-2 | NM_001293256.1 |  | P.mume.TIP1-2prob | XM_008224654.1 |
| P.mume.PIP2-5probA | XM_008235107.2 |  | P.mume.TIP1-2-like | XM_008237995.2 |
| P.mume.PIP2-5probB | XM_008245712.1 |  | P.mume.TIP1-3pred | XM_008235352.2 |
| P.avium.PIP1.1 | KR012936.1 |  | P.mume.TIP2-1pred | XM_008230247.2 |
| P.avium.PIP1.2 | KR012937.1 |  | P.mume.TIP3-2prob | XM_008242207.1 |
| P.avium.PIP1-3pred | XM_021965115.1 |  | P.mume.TIP4-1pred | XM_008221848.1 |
| P.avium.PIP1.3 | KR012938.1 |  | P.mume.TIP5-1prob | XM_016794507.1 |
| P.avium.PIP1-3-like | XM_021944645.1 |  | P.mume.TIP-type1prob | XM_008240244.2 |
| P.avium.PIP1-4prob | XM_021977323.1 |  | P.mume.TIP-type2prob | XM_008240243.1 |
| P.avium.PIP2.1 | KR012939.1 |  | P.avium.TIP1-1-like | XM_021968382.1 |
| P.avium.PIP2.2 | KR012940.1 |  | P.avium.TIP1-3pred | XM_021958018.1 |
| P.avium.PIP2-2prob | XM_021974735.1 |  | P.avium.TIP2-1pred | XM_021973489.1 |
| P.avium.PIP2.3 | KR012941.1 |  | P.avium.TIP3-2prob | XM_021946622.1 |
| P.avium.PIP2.4 | KR012942.1 |  | P.avium.TIP4-1pred | XM_021963711.1 |
| P.avium.PIP2-5probA | XM_021970894.1 |  | P.avium.TIP5-1prob | XM_021967793.1 |
| P.avium.PIP2-5probB | XM_021954392.1 |  | P.avium.TIP-type | XM_021958679.1 |
| PcXPm.PIP1.1 | KR012929.1 |  | P.persica.TIP1-1pred | XM_007202413.2 |
| PcXPm.PIP1.2 | KR012930.1 |  | P.persica.TIP1-2pred | XM_007199283.1 |
| PcXPm.PIP1.3 | KR012931.1 |  | P.persica.TIP1-2prob | XM_007205705.2 |
| PcXPm.PIP2.1 | KR012932.1 |  | P.persica.TIP1-3pred | XM_007218785.2 |
| PcXPm.PIP2.2 | KR012933.1 |  | P.persica.TIP2-1pred | XM_007215801.2 |
| PcXPm.PIP2.3 | KR012934.1 |  | P.persica.TIP3-2prob | XM_007210468.2 |
| PcXPm.PIP2.4 | KR012935.1 |  | P.persica.TIP4-1pred | XM_007225968.2 |
| P.persica.PIP1-1 | JX317646.1 |  | P.persica.TIP5-1prob | XM_007219285.1 |
| P.persica.PIP1-2pred1 | XM_007209372.2 |  | P.persica.TIP-type | XM_007209455.2 |
| P.persica.PIP1-2pred2 | XM_020556696.1 |  | P.persica.TIP1 | AB126924.1 |
| P.persica.PIP1-4prob | XM_007218706.2 |  | PdTIP1-2 | XM_034373330.1 |
| P.persica.PIP2-2 | XM_007205627.2 |  | PdTIP1-1 | XM_034367674.1 |
| P.persica.PIP2-5probA | XM_007207282.2 |  | PdTIP1-3 | XM_034363611.1 |
| P.persica.PIP2-5probB | XM_007218710.2 |  | PdTIP2-1 | XM_034354508.1 |
| PdPIP1-1 | XM_034349978.1 |  | PdTIP2-2 | XM_034358950.1 |
| PdPIP1-2 | XM_034348745.1 |  | PdTIP3-2 | XM_034359066.1 |
| PdPIP1-3 | XM_034361943.1 |  | PdTIP4-1 | XM_034344897.1 |
| PdPIP2-1 | XM_034348514.1 |  | PdTIP5-1 | XM_034349712.1 |
| PdPIP2-2 | XM_034363911.1 |  |  |  |
| PdPIP2-3 | XM_034366884.1 |  |  |  |
| PdPIP2-4 | XM_034370909.1 |  |  |  |

Shortening code: P: Prunus, PcXPm: *Prunus cerasifera* x *Prunus munsoniana*, Pd: *Prunus dulcis*, prob: probable, pred: predicted, X: transcript variant.

**Supplementary Table 3**. Primers sequences used for measurement of *Prunus dulcis* (Pd) aquaporins expression by RT-qPCR. Columns: AQP group, Name (assigned name based in homology with Prunus sp sequences in NCBI), Primer Forward (sequences in 3’-5’), Primer Reverse sequence (sequences in 3’-5’), NCBI accession.

| **AQP Group** | **Named** | **Primer Forward** | **Primer Reverse** | **NCBI accession** |
| --- | --- | --- | --- | --- |
| **PIPs1** | PdPIP1.1 | GCTCCACTTCCCATCG | GGCTCCTAGCAGGGTTG | XM_034349978.1 |
|  | PdPIP1.2 | CAAGGACTACAAGGAGCC | ACAGGAAAAGGAAGGTGG | XM_034348745.1 |
|  | PdPIP1.3 | AGGGTGGTGGTCTTGG | GAGAGTCTCTGGCATTCC | XM_034361943.1 |
| **PIPs2** | PdPIP2.1 | ACTCTTCGATGCAGTGGAGC | CAGTGACATAGAGGAAAAGAAG | XM_034348514.1 |
|  | PdPIP2.2 | GATTCTCAGGAAAAGACTACC | GTGATGTACAAGAACAAGAGG | XM_034363911.1 |
|  | PdPIP2.3 | CGTCATTGGCTACAAGTCC | GAATAGTCCAAAGGTCACAGC | XM_034366884.1 |
|  | PdPIP2.4 | GCTCGCAAGGTCTCGTTGAT | GAGTTGTAGTTGTGCTTCTGG | XM_034370909.1 |
| **TIPs1** | PdTIP1.1 | CCAACTACCAGACTACCTC | CACGCTGCCTCTCTTCG | XM_034367674.1 |
|  | PdTIP1.2 | CAGAGATGTGGAGAGCAG | ATGAGATGATTGAAGAGGTCAG | XM_034373330.1 |
|  | PdTIP1.3 | GGCTCTGGCATGGCTTT | CCAAATGTGACAGCAGG | XM_034363611.1 |
| **TIPs2** | PdTIP2.1 | ATCTTCTGCTCTCCATACTC | CCAAACTGAAAGAATCATCAAAG | XM_034354508.1 |
| **TIPs-type** | PdTIP2.2 | GTGAAGTTGGCTTTTGGTAGC | AAGGGTGGCAATGAACTCAGC | XM_034358950.1 |
| **TIPs3** | PdTIP3.2 | GCTCTGTTCTCGCACTTGG | GGTTACAGCAGGGTTGACG | XM_034359066.1 |
| **TIPs4** | PdTIP4.1 | CACTGGGTTTACTGGGTTG | AATGGGGAGATGAGTTGTTGG | XM_034344897.1 |
| **TIPs5** | PdTIP5.1 | GCAAGCAGTTACTCCCAATGC | TGGCATCAACTTTCTGGAGG | XM_034349712.1 |

Shortening code: Pd: *Prunus dulcis*, PIP: Plasma membrane intrinsic proteins, TIP: Tonoplast intrinsic proteins.
